# Supplementary material for: 8a, a New Acridine Antiproliferative and Pro-Apoptotic Agent Targeting HDAC1/DNMT1
Source: Int J Mol Sci. 2021 May 24;22(11):5516. doi: 10.3390/ijms22115516 (PMC8197214; doi:10.3390/ijms22115516)
Supplement: Supplementary file 1 [file ijms-22-05516-s001.zip › ijms-1204887-supplementary.pdf]

8a, a new acridine antiproliferative and pro-apoptotic agent lead compound targeting HDAC1/DNMT1

Qiting Zhang [1], Ziyan Wang [2], Xinyuan Chen [2], Haoxiang Qiu [2], Yifan Gu [2], Ning Wang [1,*], Tao Wang [1], Ze Wang [2], Huabin Ma [1], Yufen Zhao [1, 3, *], Bin Zhang [2,*]

1 Institute of Drug Discovery Technology, Ningbo University, Ningbo, 315211, PR China
2 Li Dak Sum Yip Yio Chin Kenneth Li Marine Biopharmaceutical Research Center, College of Food and Pharmaceutical Sciences, Ningbo University, Ningbo, 315800, PR China
3 Key Lab of Bioorganic Phosphorus Chemistry & Chemical Biology, Department of Chemistry, Tsinghua University, Beijing, 100084, P.R. China.

* Correspondence: wangning2@nbu.edu.cn (N. Wang), zhaoyufen@nbu.edu.cn (YF. Zhao), zhangbin1@nbu.edu.cn (B. Zhang)

Figure

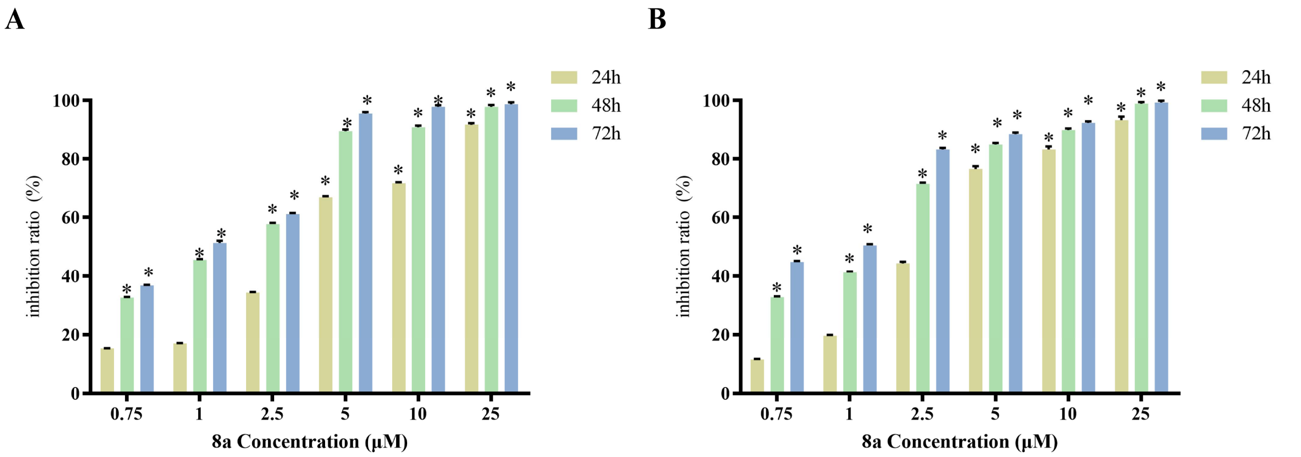

Figure S1. (**A-B**). Cell viability through use of an MTT assay. CCRF-CEM (A) and K562 (B) cells were treated with different concentrations of **8a** (0.75, 1, 2.5, 5, 10 and 25 μM) for different amounts of time (24, 48, and 72h) (* $p < 0.05$, ** $p < 0.01$).

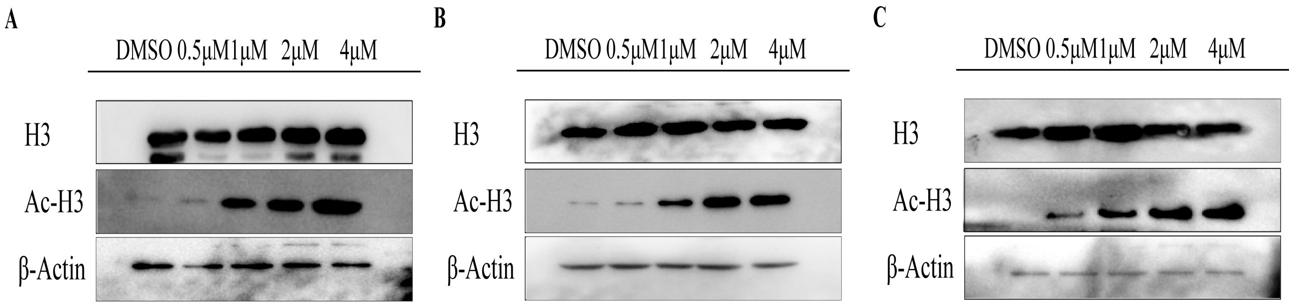

Figure S2. (A-C). Basal expression of Ac-H3 and total H3 in 8a-treated U937 (A), CCRF-CEM (B) and K562 (C) cell lines. cells were treated with different concentrations of 8a (0.5, 1,2, 5, and 10 μM) for 48 h. Protein was extracted and immuno-blotted with antibodies against H3, Ac-H3 and β-actin. 0.1%DMSO was used as a negative control. Western blots were quantified, and data are presented as means ± SD of three independent experiments (n = 3) (* $p < 0.05$, ** $p < 0.01$).

A

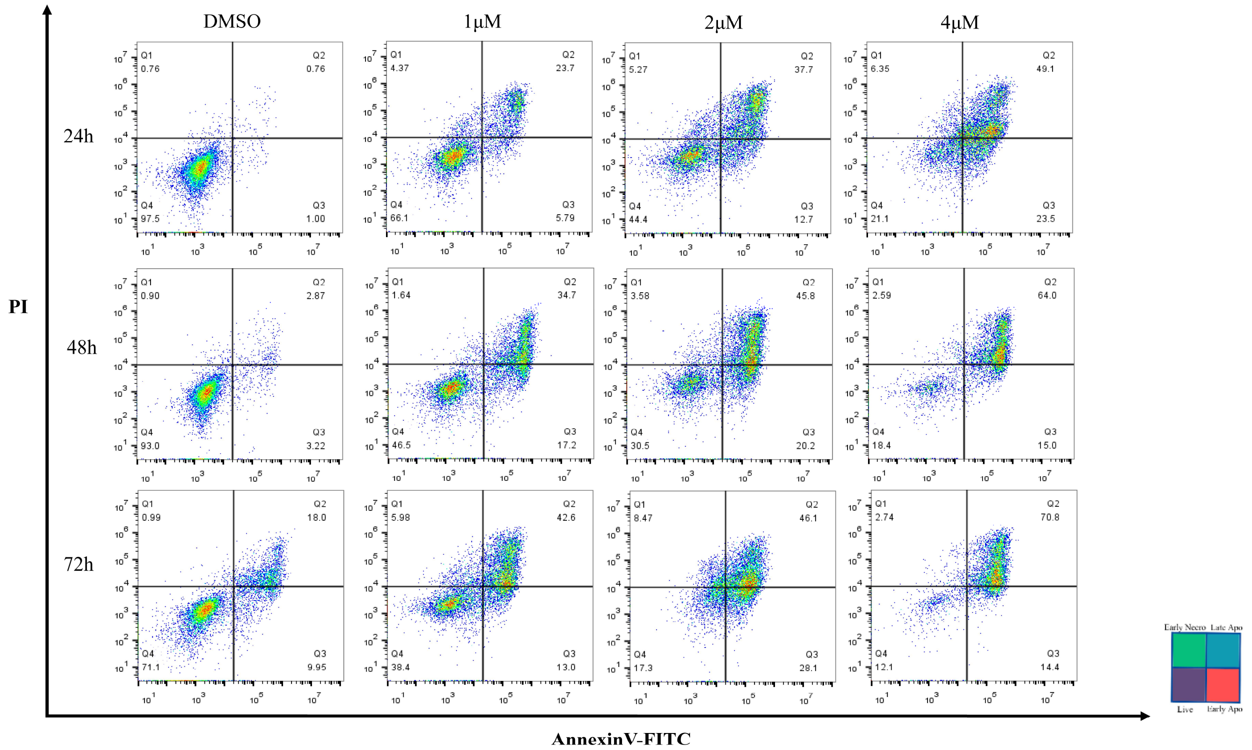

B

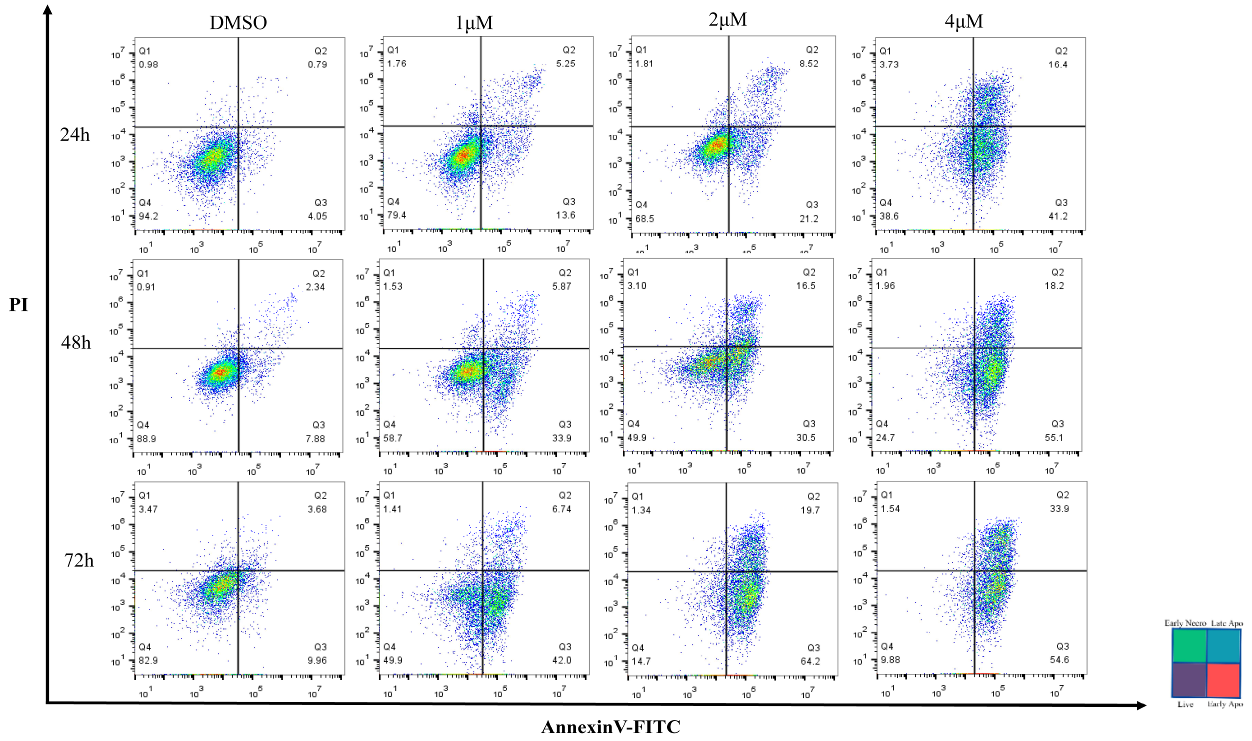

Figure S3. (**A-B**) Annexin V-FITC apoptosis assay. CCRF-CEM cells and K562 cells were treated with different concentrations of 8a (1, 2, 4μM) and at a different time (24, 48, and 72h). Propidium iodide can stain necrotic cells or cells that have lost their cell membrane integrity in the late stage of apoptosis, showing red fluorescence. Annexin V-FITC can enter the cytoplasm and bind to phosphatidylserine located on the inner side of the cell membrane, leading to the necrotic cells to present green fluorescence. 0.1%DMSO was used as a control.

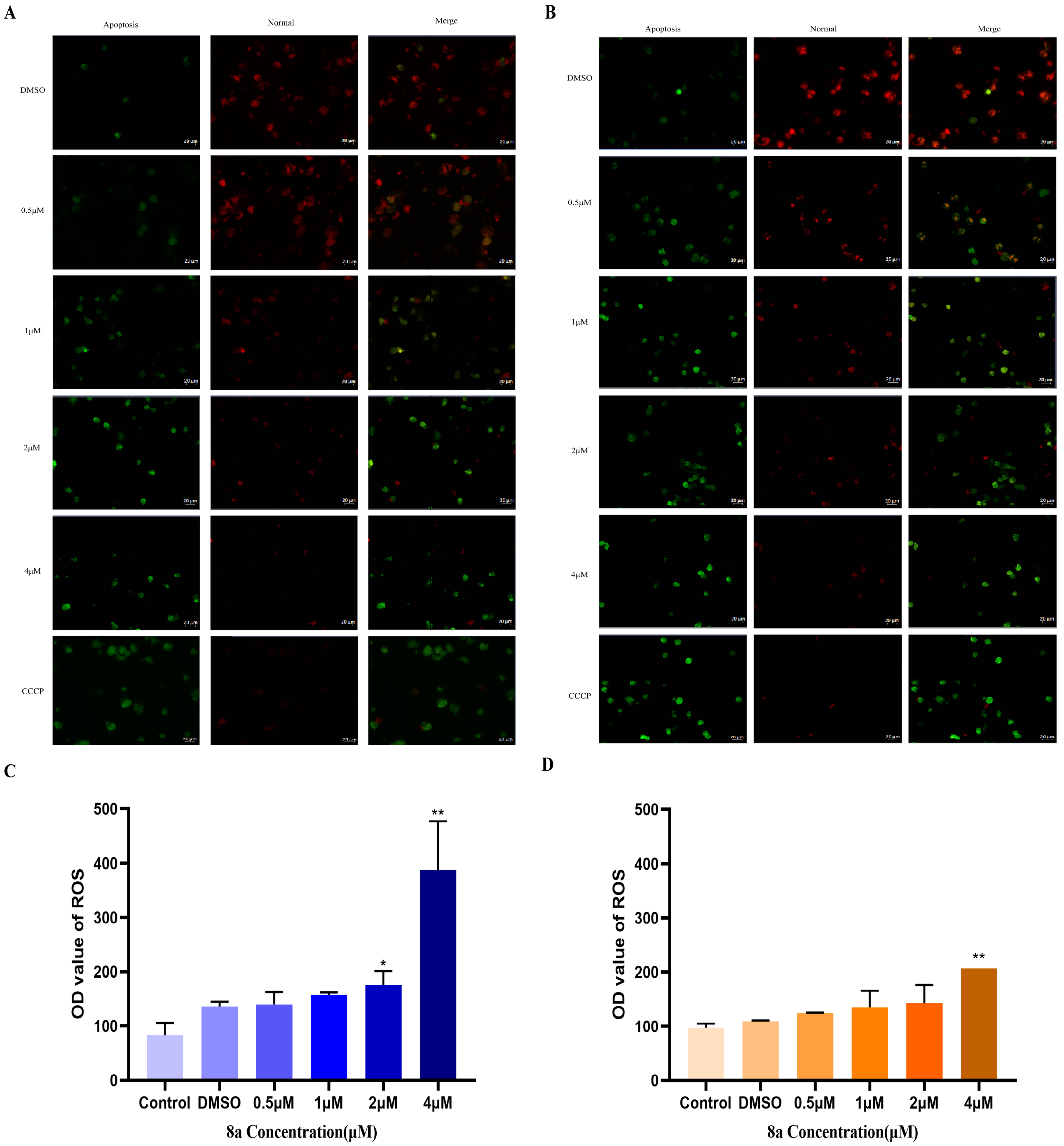

Figure S4. (**A-B**). Mitochondrial Membrane Potential. Normal cells' red fluorescence was reduced, and the green fluorescence of apoptotic cells was enhanced as the increases of concentration of 8a (0.5, 1, 2, 4μM), CCCP as a positive control, and 0.1%DMSO was used as a negative control. The normal cells showed red fluorescence, and the apoptotic cells showed green fluorescence. (**C-D**). Reactive oxygen detection. CellROX Deep Red Reagent probe was used to detect the changes of ROS in tumor cells induced by 8a, CEM and K562 cells were treated with different concentrations of 8a (0.5, 1, 2, and 4 μM) in 48h (**$p < 0.01$). 0.1%DMSO was used as a control.

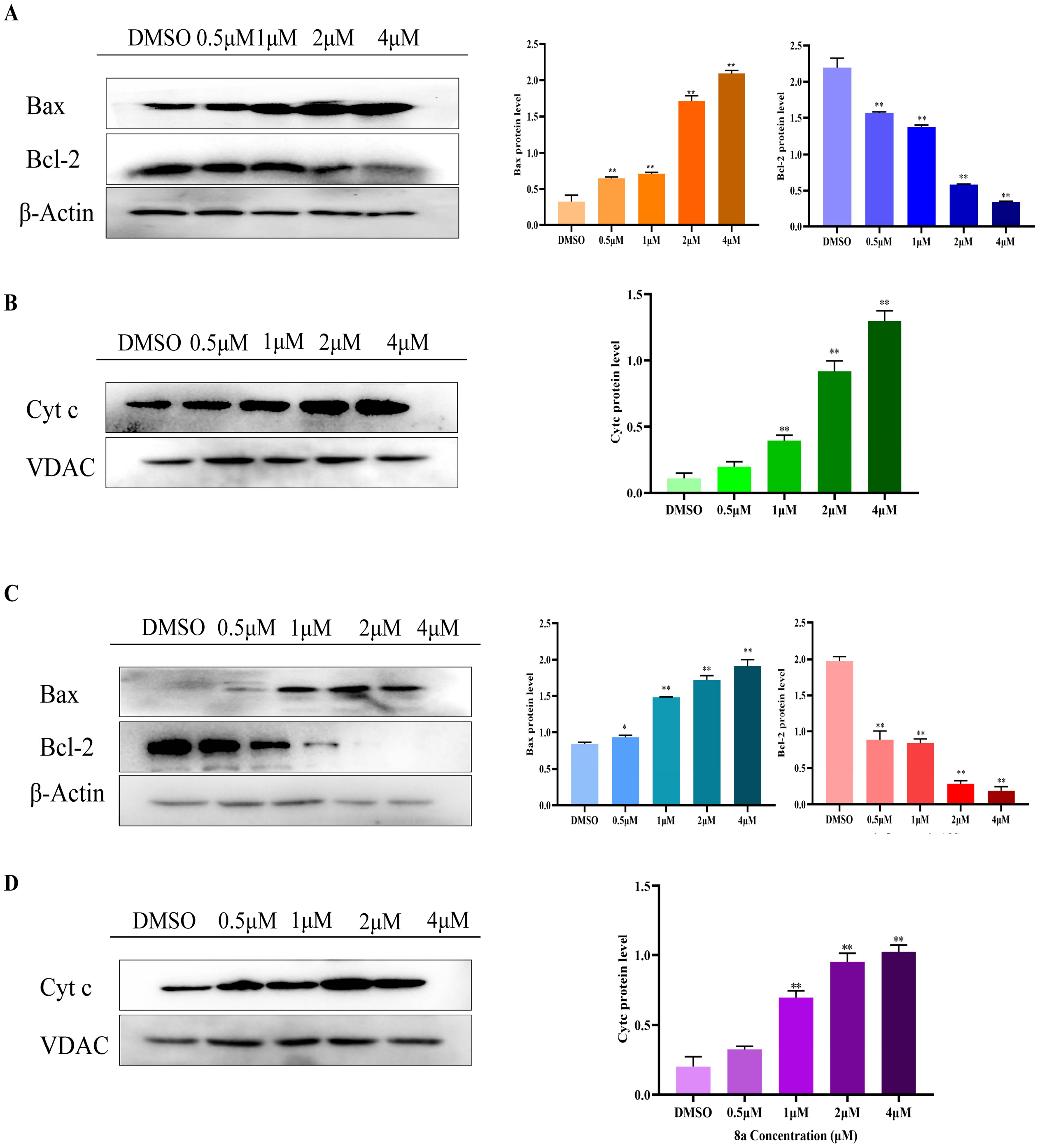

Figure S5. (**A-B**). Basal expression of apoptotic protein markers in 8a-treated CEM cell lines. CEM cells were treated with different concentrations of 8a (0.5, 1,2, 5, and 10 μM) for 48 h. (**C-D**) Basal expression of apoptotic protein markers in 8a-treated K562 cell lines. Protein was extracted and immuno-blotted with antibodies against Bax, Bcl-2, Cyt c, and β-actin. 0.1%DMSO was used as a negative control. Western blots were quantified, and data are presented as means ± SD of three independent experiments (n = 3) (* $p < 0.05$, ** $p < 0.01$).

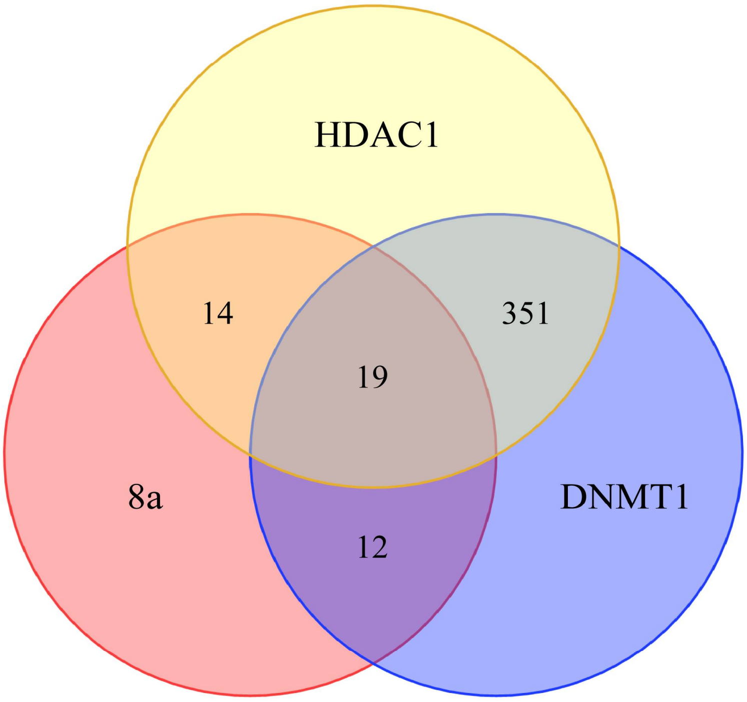

Figure S6. Venn diagram analyzes the relationship of HDAC1-related and DNMT1-related with these differential protein. There were 31 common protein between DNMT1-related protein and differential protein in the sample, and 33 common protein between HDAC1-related and differential protein in the sample. A total of 19 protien were associated with both HDAC1 and DNMT1.

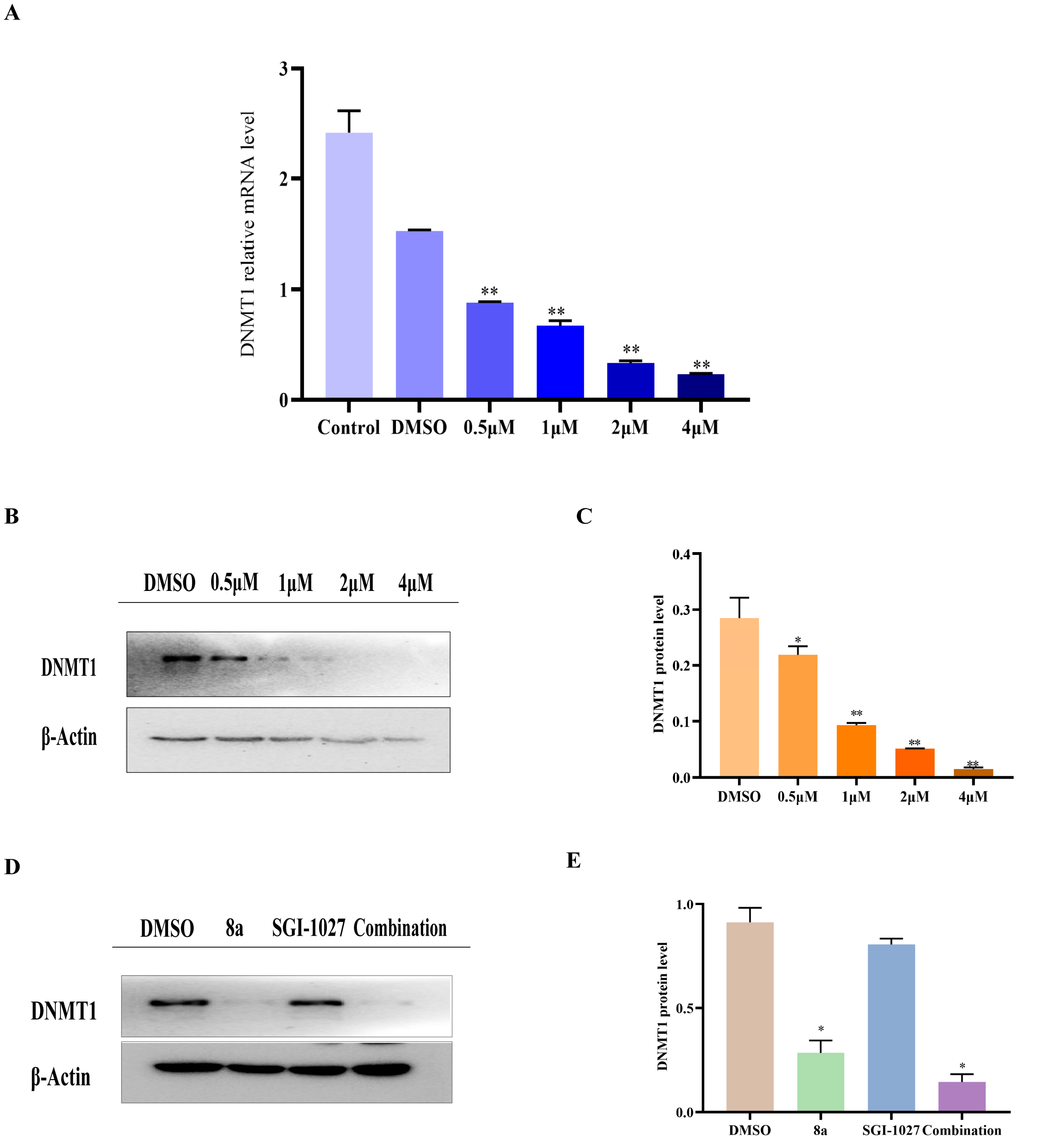

Figure S7. **8a** affected the expression of DNMT1. (**A**). RT-PCR was used to analyze the mRNA expression of *DNMT1* under different concentrations of **8a** (0.5, 1, 2, and 4 μM) in CEM cells (** $p < 0.01$). (**B-C**). Western blot analysis of whole-cell extracts from cells treated with **8a** (0.5, 1, 2, and 4 μM) for 48 h (** $p < 0.01$). 0.1%DMSO was used as the control. (**D-E**). Western blot analysis of CEM cells treated with **8a** (2 μM) and SGI-1027 (3 μM) for 48h. Whole-cell extracts from cells exposed to 0.1%DMSO was used as the controls (* $p < 0.05$, ** $p < 0.01$).

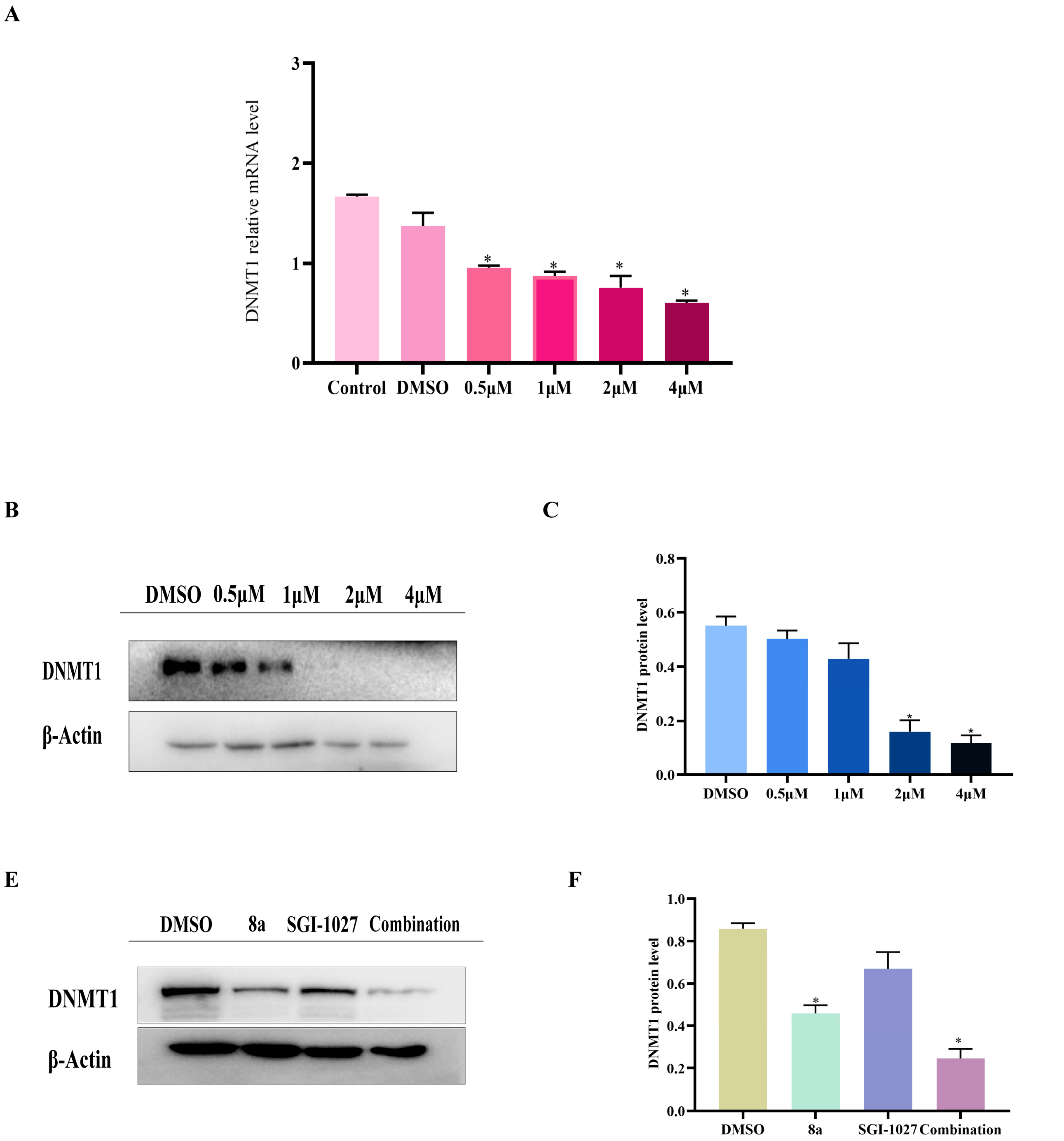

Figure S8. 8a affected the expression of DNMT1. (**A**). RT-PCR was used to analyze the mRNA expression of *DNMT1* under different concentrations of **8a** (0.5, 1, 2, and 4 μM) in K562 cells (** $p < 0.01$). (**B-C**). Western blot analysis of whole-cell extracts from cells treated with **8a** (0.5, 1, 2, and 4 μM) for 48 h (** $p < 0.01$). 0.1%DMSO was used as the control. (**D-E**). Western blot analysis of K562 cells treated with **8a** (2 μM) and SGI-1027 (3 μM) for 48h. Whole-cell extracts from cells exposed to 0.1%DMSO was used as the controls (* $p < 0.05$, ** $p < 0.01$).

Methods

4.8 Proteomics

1. Sample preparation

The cell cultivation in 6 mm plate, the number of cells about accounts for 30% ~ 40% of orifice, set up 8a concentration of 2 μM, DMSO as control group. each concentration three parallel, cultured cells for 48 h.

2. Cell lysis

a. Mix the cells thoroughly with the tip, draw into a 2 mL EP tube, centrifuge 1000g, 4℃ for 5 min.

b. Pour away the supernatant, wash with PBS (phosphate buffer saline) three times, centrifuge 1000 g at room temperature for 5 min.

c. Pour away the supernatant again, add 400 μL protein lysate containing 1xPMSF (Phenylmethanesulfonyl fluoride) protease inhibitor.

d. Ultrasound for 5 s, stop for 10 s, 3 cycles, place on ice, centrifuge 15000 g at 4℃ for 10min.

e. The supernatant was taken into the new EP tube, and the protein concentration was measured.

3. Trypsin enzymatic hydrolysis.

a. 40 uL DTT (DL-Dithiothreitol)（10 mM) were added into the Millpore10 kD tube, then 100 μg protein was added, the total volume was 500 μL (if the egg laying was less than 100 μg, the amount of DTT added was counted again, the sum of the two was 500 μL).

b. Centrifuge at 20℃ for 10 min. The filtrate was discarded each time and 400 μL DTT was added again. Repeat for three times.

c. The ultrafiltration tubes were placed in a metal bath at 50~55℃ for 20min.

d. Remove the ultrafiltration tube and place it in the original ultrafiltration device and cool to room temperature.

e. Add 400 μL IAA (indole-3-acetic acid) (20 mM), centrifuge 14000 g at 20℃ for 10min. The filtrate was discarded each time and 400 μL IAA was added again. Repeat three times.

f. Reaction at room temperature in darkness for 30 min.

h. Add 400 μL NH_4HCO_3 (50 mM) and centrifuge for 14000 g at 20℃ for 10 min. Each time the filtrate was discarded, 400 μL NH_4HCO_3 (50mM) was added again, and repeated three times.

i. Collect the protein into the new centrifuge tube (remove the ultrafiltration tube and place it upside down in the centrifuge tube); Centrifuge 1000 g at 20℃ for 1min.

j. After adding Typsin, adjust pH to 7-9, W_{Typsin}: W_{Sample} =1:50~1:20, overnight at 37℃ (12~14h).

k. 10% Trifluoroacetic acid (TFA) was added to make pH<2 and the enzymatic hydrolysis was terminated.

4. Put it in -80℃ for 1h, take it out and place it in the lyophilizer to freeze dry.

5. Remove the sample from the lyophilizer and centrifuge 12000 g for 10 min.

6. Protein demineralization (Pierce C18 tips)

a. 20 μL 5% TFA diluted sample.

b. Column activation: 10 μL 50 % Acetonitrile (ACN) two suction hits.

c. Column balance: 10 μL 0.1% TFA suction hit twice.

d. Load sample: the sample solution is adsorbed on C18 tips (loading twice).

e. Wash: 10 μL 0.1% TFA wash 5 times.

f. Elution: Add 10 0 μL 50% ACN (including 0.1%TFA) into a new PE tube.

g. Lyophilize the sample and dilute it with 10~20 μL 0.1% TFA.

h. Protein content of each sample was 500 ng, and mass spectrometry was performed.

Full-length gels and blots

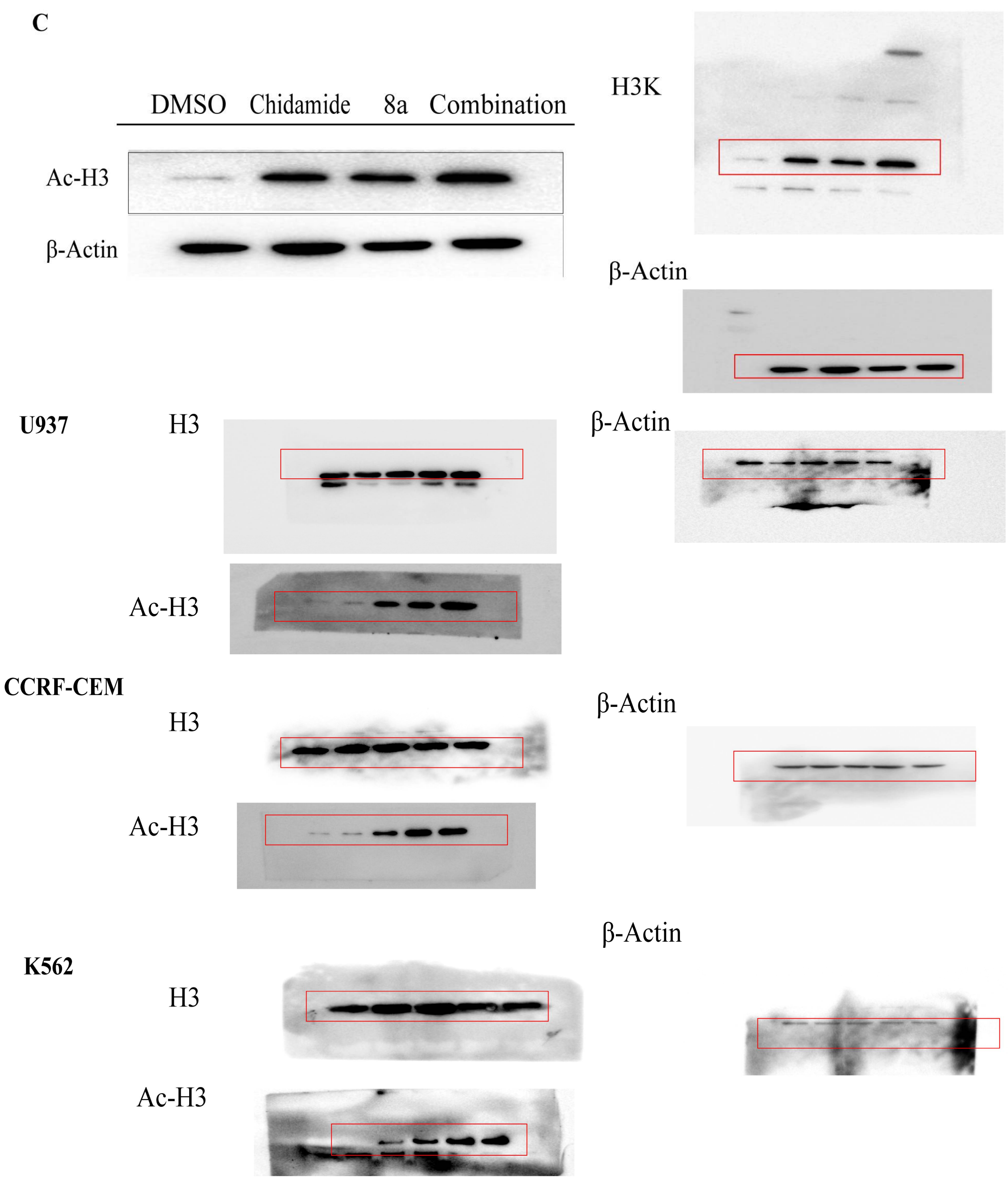

Figure 2 and Figure S2

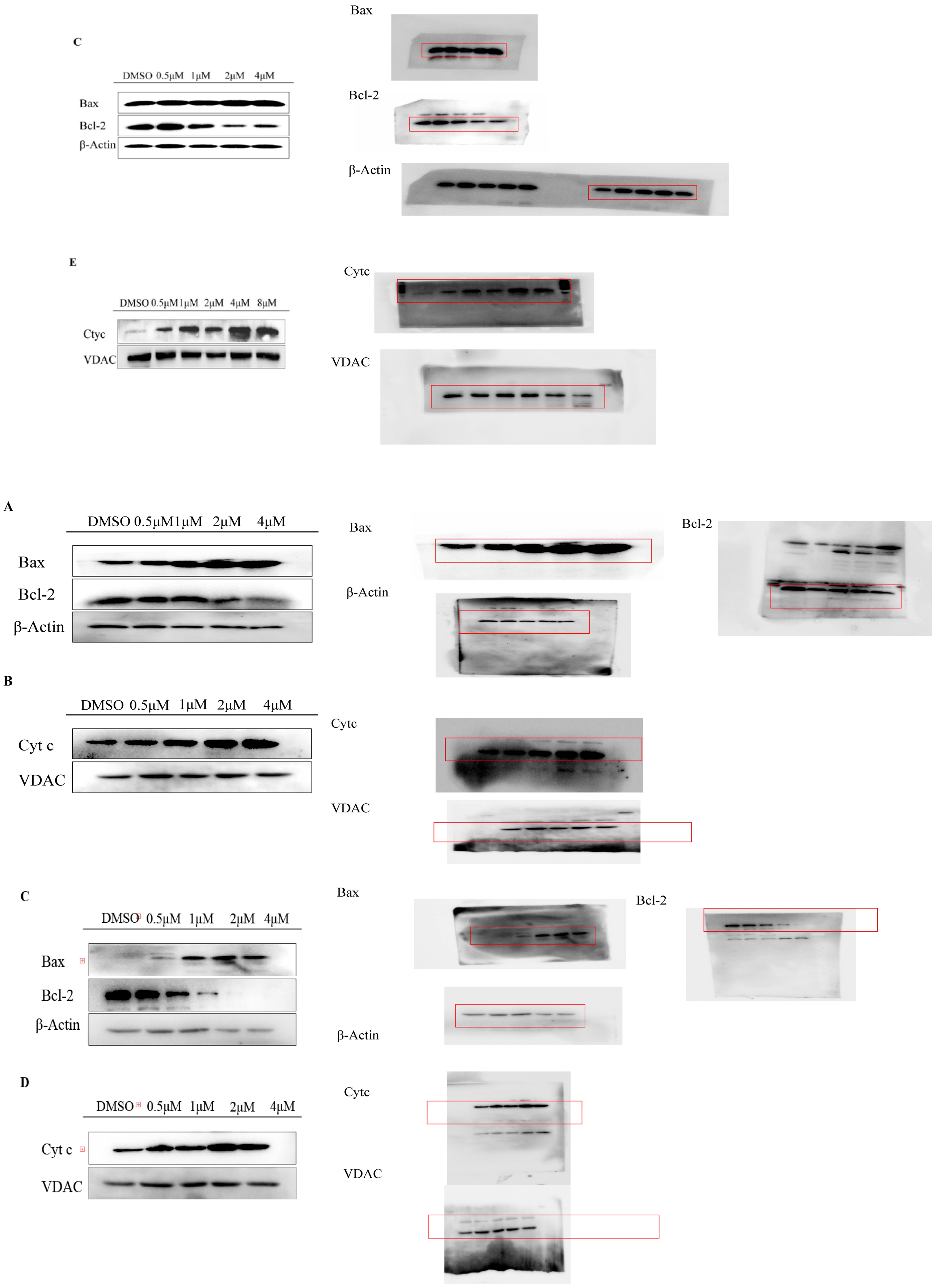

Figure 3 and figure S5

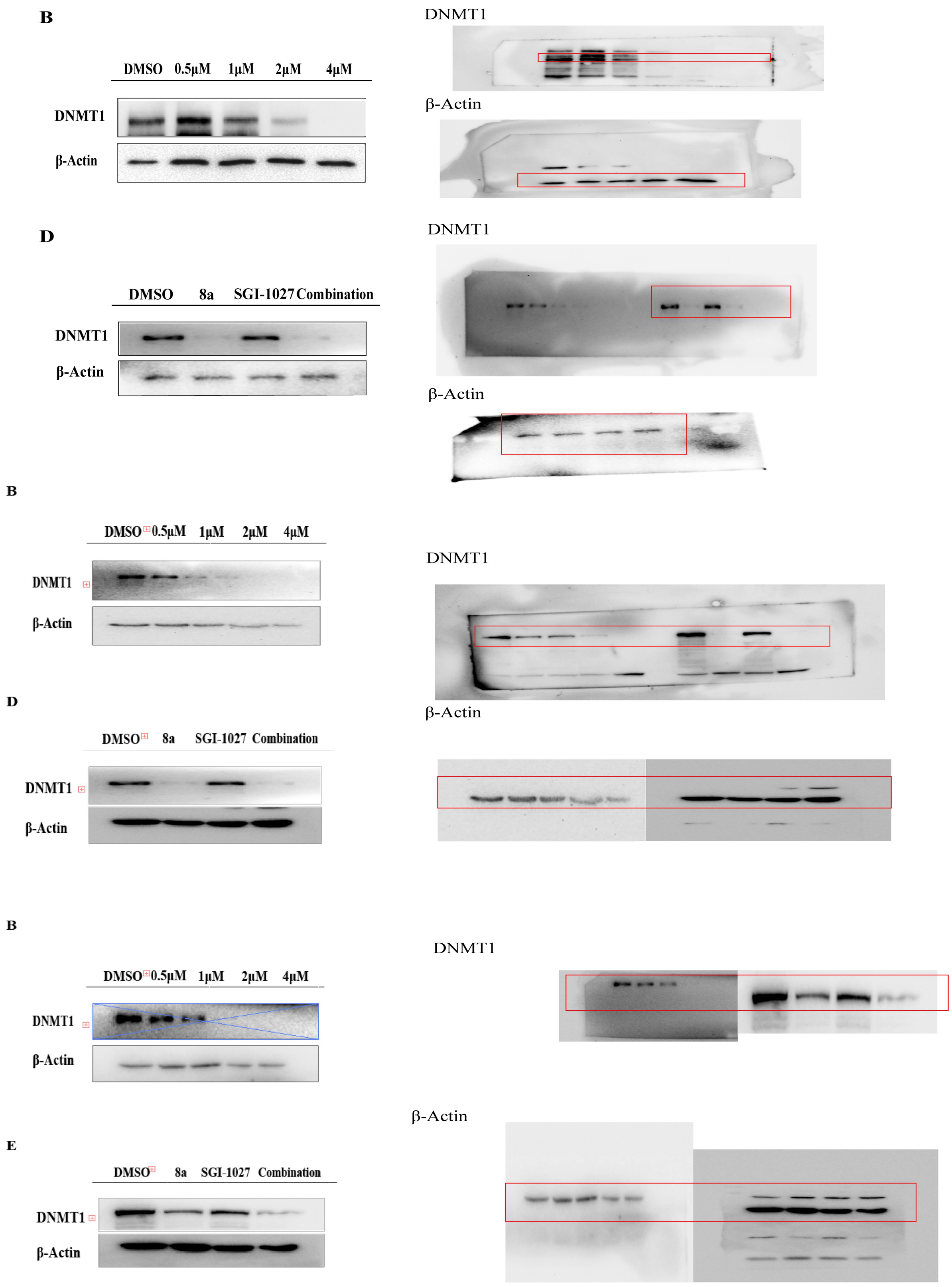

Figure 6 and figure S7-S8
